# Milk Other Than Breast Milk and the Development of Asthma in Children 3 Years of Age. A Birth Cohort Study (2006–2011)

**DOI:** 10.3390/nu10111798

**Published:** 2018-11-19

**Authors:** Asmaa El-Heneidy, Manar E. Abdel-Rahman, Gabor Mihala, Lynda J. Ross, Tracy A. Comans

**Affiliations:** 1 Centre for Applied Health Economics, School of Medicine, Menzies Health Institute Queensland, Griffith University, QLD 4111, Australia; mph.asmaa@gmail.com (A.E.-H.); g.mihala@griffith.edu.au (G.M.); t.comans@uq.edu.au (T.A.C.); 2 Department of Public Health, College of Health Sciences, Qatar University, Doha 2713, Qatar; 3 Menzies Health Institute Queensland, Griffith University, QLD 4222, Australia; lynda.ross@griffith.edu.au; 4 Centre for Health Services Research, University of Queensland, Brisbane QLD 4072, Australia

**Keywords:** asthma, breastfeeding, milk other than breast milk

## Abstract

Prevalence of asthma in Australian children is amongst the highest in the world. Although breastfeeding positively influences infant immunity, early introduction of Milk Other than Breast Milk (MOTBM) may also play an important role in the development of Asthma. The aim of this study was to investigate the association between the introduction of MOTBM in the first six months after birth and the development of reported persistent asthma in 3-year olds. A sample of 1121 children was extracted from the Environments for Healthy Living longitudinal birth cohort study. Introduction of MOTBM during the first six months after birth increased almost two-fold the risk of development of persistent asthma after adjusting for other covariates (Adjusted Relative Risk (ARR): 1.71, 95% CI: 1.03–2.83, *p* = 0.038). This study indicates that the introduction of MOTBM in the first six months of life is a risk factor for asthma incidence among 3-year old children. This result is important in explaining the benefits of breastfeeding as part of public health interventions to encourage mothers to increase breastfeeding initiation and duration, and avoid the introduction of MOTBM in the first six months after childbirth.

## 1. Introduction

Asthma is a major public health issue predominantly facing developed countries, including Australia [1]. It is the most common chronic disease among children [2]. The prevalence of asthma in Australian children is amongst the highest in the world [3]. The worldwide prevalence of childhood asthma has been increasing considerably in the last few years [4]. Explanations for this increase are not yet clear, thus limiting the opportunities to develop targeted primary prevention measures [4].

According to hygiene hypothesis, changes of lifestyle in industrialized and western countries, in combination with limited exposure to microorganisms such as viruses and bacteria during early childhood, increase the susceptibility to allergic diseases including asthma [5]. Children raised in developed cities like those in Australia are exposed to indoor and outdoor allergens and irritants during their early years of life. This may have tipped the balance of the child’s immune system from a TH1 (no asthmatic) to a TH2 (asthmatic) phenotype response [6].

Clinically, asthma is defined as a chronic inflammatory disorder of the lungs. It is characterized by recurrent episodes of wheezing, shortness of breath, tightness of chest, and coughing, associated with limitation of airflow in the respiratory system [7]. Asthma and other allergic diseases are often linked to the immune system and inflammation response. This is because the immune system is thought to be a regulator of asthma and airways inflammations by producing immune factors in response to stimuli and foreign bodies [8].

Defining asthma through testing lung function is challenging before the age of 5 [9]. Nonetheless, several studies monitored early signs of asthma during the first 3 years of the infant’s life to test for the impact of various factors on development of asthma in later life [10,11,12]. Several studies documented a number of factors that may increase the likelihood of developing asthma, like maternal asthma [13], young maternal age [14], low level of mother’s education [15], Aboriginality [16], child sex [17], maternal smoking during pregnancy [18], early gestational age [19], caesarean section [20], prematurity [21], low birth weight [22], the existence of eczema [23], and food allergies [24].

Infant feeding is an important early-life exposure that may influence the development of asthma [7]. The components of breast-milk stimulate the infant’s own immune system, safeguarding them against the development of allergic disease [7]. Although breastfeeding provides health benefits against respiratory infections and maturation of the infant’s immunity [25], findings of many studies assessing the role of breastfeeding in decreasing the risk of asthma development have been controversial [7,26,27,28]. Moreover, Milk Other than Breast Milk (MOTBM) is usually introduced early in infant life for many reasons [29], and the link between the early introduction of MOTBM and the development of Asthma has yet to be discovered.

Most infant formulas are derived from standard cow’s milk, which contains powerful food allergens [30]. There is some evidence of association between infant consumption of cow’s milk in the initial few months of life and the development of asthma [30]. Numerous studies were conducted on MOTBM feeding and its effect on development of Asthma [30,31,32,33]. These studies, however, were focusing on comparing the effects of various types of MOTBM, such as cow milk, goat milk, soymilk or hydrolyzed milk, on increasing or decreasing the risk of development of Asthma relative to each other. Some results showed that certain types of these MOTBM, such as hydrolyzed formula, reduce the risk of developing asthma [30,31,34]. However, the effect of any type of MOTBM in increasing the risk of development of Asthma relative to breast milk was not studied.

Hence, the aim of our study is to assess whether the introduction of any type of MOTBM during the first 6 months after birth increases the risk of later development of asthma among 3-year old children.

## 2. Materials and Methods

### 2.1. The Environments for Healthy Living Study

The Environments for Healthy Living study (EFHL) is a prospective, multi-level, multi-year longitudinal birth cohort study with six recruitment years from 2006 to 2011. The study was registered with the Australian New Zealand Clinical Trials Registry (ACTRN12610000931077). The study recruited pregnant women from three geographically-defined, contiguous Health Districts in Australia (Logan City and Gold Coast in Queensland, and the Tweed area in New South Wales) [35]. Perinatal data were obtained from maternal medical records following discharge from hospital completed by the hospital midwives. Data items included maternal conditions, obstetric complications, delivery information, and child information such as gender, gestational age, and birth weight [36].

The EFHL study was approved by the Griffith University Human Research Ethics Committee (Research ethics approval No is MED/16/06/HREC, MED/23/11/HREC). Additional ethical approval for participant recruitment was also obtained from each of the three participating public maternity hospitals (Logan Hospital HREC/06/QPAH/96, Gold Coast Hospital HREC/06/GCH/52, The Tweed Hospital NCAHS HREC 358N). A consent form was read and signed by each participant who agreed to release the hospital perinatal data related to the birth of her child, complete a maternal baseline questionnaire, and to individual follow-up [36].

When EFHL infants turned 1, the primary carers, predominantly the mothers, completed a questionnaire (wave 1) that included questions about demographics, household socioeconomics, child feeding and diet, child health, and development (including medically diagnosed asthma and food allergy). When the children reached 3 and 5 years of age, the primary carers were again contacted and completed similar questionnaires (wave 2 and 3).

### 2.2. Study Population

**Figure 1:**
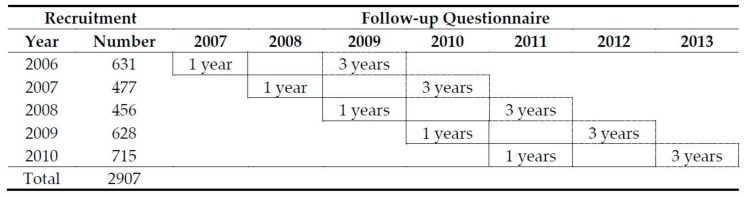
Study cohort, wave 1 (solid border) and wave 2 (dash border).

Children aged 3 from the EFHL study were included in this study. These children were recruited between 2006 and 2010 with follow-ups at 1 and 3 years of the study waves 1 and 2 respectively (Figure 1). Twins were handled as unrelated children (17 children (1.7%)).

### 2.3. Study Variables

The outcome variable, reported Medically-diagnosed persistent asthma, was derived from a question to the carer on whether the child has ever been diagnosed by a medical doctor with wheezing or asthma. A child was considered to have medically-diagnosed persistent asthma if the carer indicated that the child was diagnosed with asthma at both the 1 (wave 1) and 3 years of age (wave 2) follow-ups. Since the outcome variable focused on persistent asthma only, children whose asthma status was not constant at both waves were not considered to have persistent asthma, and thus, were excluded from the study.

The main exposure variable is the introduction of MOTBM at less than six months after birth, derived from the question: “How old was your child when he/she first had any other type of milk in a cup or a bottle? (e.g., formula, cow’s milk, soy milk)”.

Covariates were selected from the literature [13,14,15,16,17,18,19,20,21,22,23,24]. Those related to the child included: whether stopped breastfeeding at <6 months of age, gender, delivered by caesarean section, low birth weight (weight < 2500 g), preterm delivery (gestational age < 37 weeks), frequency of exposure to cigarettes smoke (derived from the question: “Indicate how regularly the baby is in a room or enclosed place where other people are smoking”), ever been diagnosed with eczema, and ever diagnosed with food allergy. Those related to the mother were: age in years, level of education, place of birth, indigenous status, average number of cigarettes smoked each day on average at >20 weeks of pregnancy and treatment of asthma during pregnancy.

### 2.4. Statistical Analyses

Medically-diagnosed persistent asthma was analyzed as a binary outcome variable. Percentages, means and standard deviations were used to describe the data. Logistic regression analyses were used to model the risk of medically-diagnosed persistent asthma in relation to introduction of MOTBM at less than six months after birth and other potential confounding variables. Purposeful selection method was used to build the models. Initially, univariate logistic regression analyses were performed to select variables to be included in the multivariable logistic regression analyses. Variables with *p*-values < 0.25 were considered to develop an initial reduced model [37]. Using adjusted Wald tests, variables that tested insignificant (with *p*-values > 0.05) were then eliminated from this model. Insignificant variables were included one at a time, and assessed for significance and for confounding effects using a 10 percent [38] change in the coefficients. Likelihood ratio tests were used to compare models. Variance inflation factors were used to assess multicollinearity among variables. Hosmer and Lemeshow goodness-of-fit tests [39] were used to assess goodness of fit of the final model. With a low incidence of the outcome, Relative Risks (RR), Adjusted Relative Risks (ARR) and their 95% confidence intervals were approximated from the corresponding Odds Ratios (OR) obtained from the logistic regression analyses. STATA 15.0 was used for all analyses [40].

## 3. Results

Information was obtained on 2907 children whose mothers were enrolled in the study from 2006 to 2010. About 51% of the children were lost to follow-up either at waves 1 or 2, or had incomplete mothers’ perinatal data. A total of 1342 children had complete information on reported medical diagnosis of asthma at the 1 year (wave 1) and 3 years (wave 2) follow-ups. Children who had consistently reported medically-diagnosed persistent asthma at both waves were included in the study, as well as those who had not (*n* = 1138). Those whose asthma status was not constant at both waves were excluded (*n* = 204). Out of the 1138 children, 17 with missing information on MOTBM were also excluded. The analytical study cohort became 1121 children.

### 3.1. Characteristics of the Study Cohort

Among 1121 children, about 7% of children had persistent asthma at the age of 3, and 51% received MOTBM at less than six months after birth. There were slightly more females (51.7%) than males (47.8%). About one-third of the children (31.2%) were delivered by caesarean section, and most were born full term (97.8%) and had normal birth weight (97.9%). Sixty-two percent of the mothers were aged 26–35 years, and only a quarter completed university (24.8). Mothers were mostly born in Australia (72.2%), not Aboriginal/TSI (96.5%), and did not smoke cigarettes each day during pregnancy (89.2%). Key characteristics of the cohort are detailed in Table 1 and Table 2.

There were some differences in the characteristics of the study children when compared to the excluded children who were either lost to follow-up (51%, *n* = 1484) or had missing information (10%, *n* = 302). Mothers in the study sample were older (51.2% were 31+ years old) than those in the excluded sample (35.7% were 31+ years) (*p* < 0.001). They were also more educated, with 85.6% of them completing at least high school compared to 75.1% in the excluded sample (*p* < 0.001). Cigarettes smoking during pregnancy was less prevalent in the study sample (4.8%) than in the excluded sample (10%) (*p* < 0.001). There were more females (52%) in the study sample than in the excluded sample (47.7%) (*p* = 0.025). More children were delivered by caesarean section (31.3%) in the study sample than in the excluded sample (27%) (*p* = 0.013). There was also a lower representation of preterm children in the study sample (2.3%), versus the excluded sample (3.9%) (*p* = 0.023). Fewer children in the study sample (6.3%) were sometimes exposed to cigarettes smoke than in the excluded sample (9.4%) (*p* = 0.012). Moreover, less children in the study sample had MOTBM at less than six months after birth (51%) compared to the excluded sample (61.2%) (*p* < 0.001). All other characteristics were not significantly different.

### 3.2. Association between Developing Persistent Asthma and MOTBM and Other Covariates

Table 1 and Table 2 also report the associations between developing persistent asthma and MOTBM and other covariates. More children who were introduced to MOTBM at less than six months after birth (8.7%) had persistent asthma compared to those who were not introduced (4.7%). Persistent asthma was much more prevalent among males (10.4%) compared to females (3.4%). Younger (16–25 years) (10.9%) and less educated mothers (8.0%) had higher percentages of children with persistent asthma than older ones (5.9%) and mothers who completed university (2.9%) respectively. Although only 10% of children were ever diagnosed with eczema, the diagnosis of persistent asthma was almost twice in this group (13.3%) compared to those who were not diagnosed with eczema (6.1%). A similar pattern was seen for children who had been diagnosed with a food allergy. The percentage of children with persistent asthma was much higher among those who were sometimes exposed to cigarettes smoke (11.3%) than those who were never exposed to smoke (6.5%). Moreover, the level of passive smoking based on the total number of cigarettes smoked by mothers during their pregnancies per day did not show a dose-response effect. Persistent asthma was recorded in 18.9% of children who were exposed to tobacco smoke of more than 10 cigarettes/day, while this percentage was about 6% for both children of mothers who smoked less than 10 cigarettes/day or did not smoke at all.

Table 3 summarizes the results of univariate and multivariable logistic regression modeling of the risk of medically-diagnosed persistent asthma in relation to other variables. Univariate analysis did not reveal any associations with child cesarean section delivery, low birth weight, preterm delivery, whether child stopped breastfeeding at less than 6 months, or the frequency at which the child was exposed to cigarette smoke. Although the mother’s level of education was significant in the univariate analysis, it became insignificant in the final built multivariable model. The final model demonstrated that introduction of MOTBM in the first 6 months of life increased the risk of children having persistent asthma by 3 years of age by 71% after adjusting for other covariates (ARR: 1.71, 95% CI: 1.03–2.83, *p*-value = 0.038). Children diagnosed with eczema, being male, and whose mothers’ smoked during pregnancy were associated with an increased risk of persistent asthma in children at the age of 3 after adjusting for other covariates. Although maternal age was significantly associated with persistent asthma in children (*p* < 0.001), the direction of the relationship was not consistent. Women in the 16–25 and 31–35 age groups were at higher risk compared 26–30 and 36–45 age groups.

## 4. Discussion

All screened studies compared the effects of different types of MOTBM on increasing or decreasing the risk of asthma; however, all of these studies compared the effects of different types of MOTBM relative to each other. To our knowledge, this research is the first to assess whether MOTBM, regardless of its source, increases the risk of development of asthma.

There are three major types of infant formulas: standard cow’s milk-based formulas, soy-based formulas, and hypoallergenic formulas (hydrolysed cow’s milk formulas) [41]. Previous studies assessing standard cow-based formulas found that these formulas were not protective against asthma during infancy or childhood [30,42]. Similarly, soy-based formula did not reduce the risk of asthma when compared with standard cows-based milk formula in infants and children [30]. The literature evidence supporting the preventive effects of hydrolysed infant formulas for asthma is inconsistent and insufficient. Some studies suggest that certain partially- or extensively-hydrolysed formulas may reduce the risk of asthma compared to non-hydrolysed formulas for children with a family history of atopic disease [30,42,43]. In contrast, it was reported by others that infants who received hydrolysed cow’s milk formula did not have a lower risk of asthma compared with those who received human breast milk or standard cow’s milk formula [31].

Although several studies examined the association between different infant formulas and asthma [30,31,34,42,44], this study uniquely distinguished between breast milk and any type of MOTBM. Our results revealed that in comparison with infants who received breast milk only, those who received MOTBM during the first 6 months after birth had a 71% increased risk of persistent asthma at the age of 3 (ARR: 1.71, 95% CI: 1.03–2.83, *p* = 0.038). This association was independent of established maternal and environmental risk factors.

Previous findings regarding duration of breastfeeding and the risk of asthma are contradictory. Although several studies showed that breastfeeding prevents asthma [45,46], there were also several disagreeing studies [26,47]. This study found no significant association between duration of breastfeeding up to 6 months and development of persistent asthma in the first 3 years of life (RR: 1.13, 95% CI: 0.70–1.83). This finding is consistent with previous studies carried out in Denmark and Sweden, which have shown that breastfeeding does not lower the risk of childhood asthma (OR: 1.08, 95% CI: 0.93–1.25) and (OR: 0.99, 95% CI: 0.96–1.02) respectively [47,48].

Gender appears to be a factor in the development of asthma, i.e., boys in general are reported to have more severe asthma than girls [49]. This was suggested to be the result of smaller airway diameters relative to lung volume in boys compared to girls [50,51]. Consistent to what was observed in these studies [49,52] more males (10.4%) than females (3.4%) in our study were diagnosed with persistent asthma with strong association between sex differences and development of asthma (*p*-value < 0.001).

There is evidence suggests the existence of a complex relationship between eczema and asthma. Research found that the majority of infants with early eczema develop asthma in childhood; an example of the so-called “atopic march” [23]. Researchers suggested that understanding the relationship between eczema and asthma might help in preventing asthma developing in these vulnerable children, i.e., to prevent the atopic march. Demehri and his colleagues reported the existence of many theories about the link between eczema and asthma. One theory suggests that the impairment of skin, which is body’s protective external barrier, might stimulate the immune system to over-react to any potential allergen present in the body, including the surface of the airways in the lungs [53]. In line with several previous studies [23,54,55], we found that eczema in infancy was strongly associated with the development of asthma in children at the age of 3. Multivariable analyses showed that existence of eczema at baseline increased the risk of developing asthma by about two-fold (RR: 2.15, 95% CI: 1.15–4.04, *p* = 0.017) relative to children without eczema at the beginning of the study.

Maternal smoking during pregnancy has been consistently associated with early childhood asthma [56,57]. Passive fetal exposure to tobacco smoke during pregnancy has been reported by numerous studies to result in reduced lung function in the early months of infant’s life, with a dose–response relationship between exposure and decreased airway calibre in early life [58,59]. In our study, there was a strong association between smoking during pregnancy and the development of childhood asthma, indicative of a dose response relationship. Children who were exposed to high level of tobacco smoke of more than 10 cigarettes/day had higher risk for development of asthma (ARR: 4.23, 95% CI: 1.91–9.37, *p* < 0.001 for test for trend). These results are consistent with what was previously reported by Tariq et al. [57], where the authors found that smoking during pregnancy was associated with the development of childhood asthma at two years of age (RR: 2.2, 95% CI: 1.5–3.4, *p* < 0.001).

In our study, young maternal age at delivery was also associated with an increased risk of asthma in three-year old children. This finding is consistent with previous studies conducted in United Kingdom and Finland [7,12]. Additionally, the 31–35 age group was also associated with an increased risk of asthma in children. Including age in its continuous form in the final model gave a RR = 0.95 with 95% CI: 0.91–0.99 (*p* = 0.036). This agrees with previous studies relating young maternal age with increased risk of asthma irrespective of the weak effect size.

As demonstrated by several studies [13,19], maternal asthma was also associated with an increased risk of childhood asthma. Inconsistently, our univariate analysis did not show any such association in three-year old children. It is worth mentioning that maternal asthma was not included in our multivariable analysis as the variable regarding this issue was asked only to QLD state participants. It has been hypothesized that caesarean section might increase the risk for developing asthma compared to vaginal delivery, as a result of depriving the newborn of exposure to maternal microflora [60]. A number of studies found that caesarean section might have a positive association with development of asthma [60,61]. In the present study, we did not observe similar associations. Researchers argue that mothers with lower levels of education receive limited social support and may have decreased access to health information. Overall, this increases the exposure to risk factors that affects mothers, and consequently, their children’s health negatively [15,62]. Our built multivariable model did not include maternal education as risk factor for development of asthma although it was a risk factor in the univariate analysis.

There are several limitations of this study that are consistent with biases likely to arise from cohort studies. Firstly, the recruitment was through three public maternity hospitals only in the participating districts (Logan, Gold Coast and the Tweed Hospitals) limiting the generalizability in other populations. As children included in the study were offspring of women who planned to give birth in one of these hospitals, there is a potential for selection bias. Secondly, this study has a relative low follow-up rate (49%) naturally stemming from the original EFHL study. The systematic differences between the characteristics of children remaining in the study and those lost to follow-up are likely to introduce bias. Thirdly, the use of self-report questionnaires to collect the information from primary carers may lead to recall bias as evidenced in other cohort studies [27,63]. Lastly, the outcome, medically-diagnosed asthma was addressed by a question asked at both waves 1 and 2. As the question was based on information reported by the carer, there is possibility that it does not accurately predict medically-diagnosed asthma in children. Moreover, asthma was medically diagnosed among children less than 3 years of age; this study acknowledges the challenges of such diagnosis before the age of 5 [9]. Similarly, there is a possibility of misclassification of the exposure variable, MOTBM at less than six months after birth. All these limitations may lead to imprecise risk estimation.

The strengths of this study lie in its large sample size, its prospective design, and the use of carefully constructed questionnaires to maximize accuracy and completeness through asking specific questions, which decreased recall bias of results. In addition, asthma was diagnosed by a physician and not by parental self-report of symptoms.

In summary, the current study indicates that the introduction of MOTBM during the first six months after birth is a risk factor for asthma in three-year old children. Although more studies and further analyses are needed to confirm these findings and to better understand the underlying mechanisms, public health interventions promoting the risk of early introduction of MOTBM and encouraging mothers to refrain from using it during the first six months after birth may help in reducing the morbidity and prevalence of childhood asthma. In support of previous research, the current data did not show a correlation between breastfeeding and protection against asthma.

## Figures and Tables

**Table 1:** Characteristics of children: overall and those with persistent asthma at end of follow-up.

	Total	Persistent Asthma		Total
	%	No.	%	
**Total**	100.0	76	6.8	1121
**MOTBM introduced at <6 months**				
No	49.0	26	4.7	549
Yes	51.0	50	8.7	572
**Stopped breastfeeding at <6 months**				
No	39.5	28	6.3	443
Yes	60.5	48	7.1	678
**Gender**				
Male	47.8	56	10.4	536
Female	51.7	20	3.4	580
**Delivered by caesarean section ^1^**				
No	68.4	49	7.7	767
Yes	31.2	27	6.4	350
**Low birth weight (Weight < 2500 g)**				
No	97.9	75	6.8	1098
Yes	2.1	1	4.3	23
**Preterm delivery (Gestational age < 37 weeks)**				
No	97.7	73	6.7	1095
Yes	2.3	3	11.5	26
**Frequency exposed to cigarettes smoke ^1^**				
Sometimes	6.3	8	11.3	71
Not at all	93.6	68	6.5	1049
**Ever been diagnosed with eczema**				
No	89.9	61	6.1	1008
Yes	10.1	15	13.3	113
**Even been diagnosed with food allergy**				
No	95.7	71	6.6	1073
Yes	4.3	5	10.4	48

^1^ 0.1–0.4% missing data.

**Table 2:** Characteristics of mothers: overall and those with children with persistent asthma at end of follow-up.

	Total	Persistent Asthma		Total
	%	No.	%	No.
**Age in years**				
16–25	17.9	22	10.9	201
26–30	30.9	19	5.5	346
31–35	31.1	26	7.4	349
36–45	20.1	9	4.0	225
**Highest level of education ^1^**				
Did not complete high school	14.4	12	7.5	161
Completed high school	29.6	28	8.4	332
TAFE/trade or apprenticeship	30.8	27	7.8	345
University degree	24.8	8	2.9	278
**Place of birth**				
Elsewhere	27.8	18	5.8	312
Australia	72.2	58	7.2	809
**Indigenous status ^2^**				
Aboriginal/TSI	1.4	0	0.0	16
Otherwise	96.5	75	6.9	1082
**Average number of cigarettes smoked each day during pregnancy ^1^**				
0	89.2	62	6.2	1000
≤10	5.4	4	6.6	61
>10	4.7	10	18.9	53
**Treatment for asthma during pregnancy (*n* = 864) ^3^**				
No	75.4	66	7.9	839
Yes	2.2	4	16	25

^1^ 0.4–0.6% missing data, ^2^ 2% missing data. ^3^ Question asked only to QLD state participants.

**Table 3:** Univariate and multivariable Logistic regression modeling the likelihood of medically-diagnosed persistent asthma.

	Univariate Analysis		Multivariable Analysis	
	RR [95% CI]	*p*-Value ^1^	ARR [95% CI]	*p*-Value ^2^
**MOTBM introduced at <6 months**		0.009		
No	1.00		1.00	
Yes	1.93 [1.18, 3.14]		1.71 [1.03, 2.83]	0.038
**Stopped breastfeeding at <6 months**		0.621		
No	1.00			
Yes	1.13 [0.70, 1.83]			
**Gender**		<0.001		
Male	1.00		1.00	
Female	0.31 [0.18, 0.52]		0.31 [0.18, 0.53]	0.000
**Delivered by caesarean section**		0.415		
No	1.00			
Yes	1.22 [0.75, 1.99]			
**Low birth weight (Weight < 2500 g)**		0.642		
No	1.00			
Yes	0.62 [0.08, 4.66]			
**Preterm delivery (Gestational age < 37 weeks)**		0.336		
No	1.00			
Yes	1.83 [0.54, 6.22]			
**Frequency exposed to cigarettes smoke**		0.126		
Sometimes	1.00			
Not at all	0.55 [0.25, 1.19]			
**Ever been diagnosed with eczema**		0.005		
No	1.00		1.00	
Yes	2.38 [1.30, 4.34]		2.15 [1.15, 4.04]	0.017
**Even been diagnosed with food allergy**		0.310		
No	1.00			
Yes	1.64 [0.63, 4.27]			
**Age in years**		0.028		
16–25	1.00		1.00	
26–30	0.47 [0.25, 0.90]		0.47 [0.24, 0.92]	0.028
31–35	0.65 [0.36, 1.19]		0.67 [0.36, 1.25]	0.206
36–45	0.34 [0.15, 0.75]		0.33 [0.15, 0.75]	0.009
**Highest level of education**		0.015		
Did not complete high school	1.00			
Completed high school	1.14 [0.57, 2.31]			
TAFE/trade or apprenticeship	1.05 [0.52, 2.14]			
University degree	0.37 [0.15, 0.92]			
**Place of birth**		0.404		
Elsewhere	1.00			
Australia	1.26 [0.73, 2.18]			
**Average number of cigarettes smoked each day during pregnancy**		0.001 ^3^		<0.001 ^3^
0	1.00		1.00	
≤10	1.06 [0.37, 3.02]		0.89 [0.30, 2.61]	0.831
>10	3.52 [1.69, 7.33]		4.23 [1.91, 9.37]	<0.001
**Treatment for asthma during pregnancy (*n* = 864) ^4^**		0.004	-	-
No	1.00		-	-
Yes	2.23 [0.74, 6.69]		-	-

^1^ Likelihood ratio *p*-value; ^2^ Wald test *p*-value; ^3^ Test for trend; ^4^ Question asked only to QLD state participants (not included in multivariable analysis); Multivariable analysis: RR are approximated by OR. Hosmer and Lemeshow goodness and fit test *p*-value = 0.929, 93% correct classification.
